# Supplementary material for: Tunable hydrogel-based micropillar arrays for myelination studies
Source: Nat Methods. 2026 Mar 30;23(4):854–64. doi: 10.1038/s41592-026-03048-3 (PMC13076230; doi:10.1038/s41592-026-03048-3)
Supplement: Supplementary file 1 — Supplementary Tables 1–6. [file 41592_2026_3048_MOESM1_ESM.pdf]

nature methods

Supplementary information https://doi.org/10.1038/s41592-026-03048-3

Tunable hydrogel-based micropillar arrays for myelination studies

In the format provided by the authors and unedited

Panel	Plot	Df	Comparison	Exact P-value	Summary
d	3 µm diameter	44	Flat vs 5 µm	5.76E-11	****
d	3 µm diameter	44	Flat vs 10 µm	1.56E-11	****
d	3 µm diameter	44	Flat vs 15 µm	1.29E-09	****
d	3 µm diameter	44	5 vs 10	7.10E-01	ns
d	3 µm diameter	44	5 vs 15	7.24E-01	ns
d	3 µm diameter	44	10 vs 15	1.65E-01	ns
d	5 µm diameter	44	Flat vs 5 µm	1.40E-11	****
d	5 µm diameter	44	Flat vs 10 µm	1.40E-11	****
d	5 µm diameter	44	Flat vs 15 µm	1.40E-11	****
d	5 µm diameter	44	5 vs 10	0.9999	ns
d	5 µm diameter	44	5 vs 15	0.7112	ns
d	5 µm diameter	44	10 vs 15	0.7276	ns
d	10 µm diameter	44	Flat vs 5 µm	6.12E-09	****
d	10 µm diameter	44	Flat vs 10 µm	9.16E-11	****
d	10 µm diameter	44	Flat vs 15 µm	1.33E-09	****
d	10 µm diameter	44	5 vs 10	0.5448	ns
d	10 µm diameter	44	5 vs 15	0.9665	ns
d	10 µm diameter	44	10 vs 15	0.8188	ns
f	3 µm diameter	21	5 µm vs 10 µm	0.00920	**
f	3 µm diameter	21	5 µm vs 15 µm	0.00020	***
f	3 µm diameter	21	10 µm vs 15 µm	0.2651	ns
f	5 µm diameter	21	5 µm vs 10 µm	0.01240	*
f	5 µm diameter	21	5 µm vs 15 µm	0.03170	*
f	5 µm diameter	21	10 µm vs 15 µm	0.9046	ns
f	10 µm diameter	21	5 µm vs 10 µm	0.60190	ns
f	10 µm diameter	21	5 µm vs 15 µm	0.10670	ns
f	10 µm diameter	21	10 µm vs 15 µm	0.48750	ns
f	5 µm distance	21	3 µm vs 5 µm	0.01877	*
f	5 µm distance	21	3 µm vs 10 µm	0.00000	****
f	5 µm distance	21	5 µm vs 10 µm	0.00373	**
f	10 µm distance	21	3 µm vs 5 µm	0.00360	**
f	10 µm distance	21	3 µm vs 10 µm	0.00010	***
f	10 µm distance	21	5 µm vs 10 µm	0.302	ns
f	15 µm distance	21	3 µm vs 5 µm	0.4623	ns
f	15 µm distance	21	3 µm vs 10 µm	0.00196	**
f	15 µm distance	21	5 µm vs 10 µm	0.03035	*
g	5 µm distance	15	3 µm vs 5 µm	4.97E-07	****
g	5 µm distance	15	3 µm vs 10 µm	1.90E-10	****
g	5 µm distance	15	5 µm vs 10 µm	0.00001103	****

Supplementary Table 1: Statistics derived from Fig. 3. One-way ANOVA with Tukey's tests were employed, *p < 0.05, **p < 0.01, and ***p < 0.001, ****p<0.0001.

Panel	Plot	Df	Comparison	Exact P-value	Summary
b	5 µm diameter cell number	6	ultrasoft vs soft	0.9327	ns
b	5 µm diameter cell number	6	ultrasoft vs stiff	0.8431	ns
b	5 µm diameter cell number	6	soft vs stiff	0.6473	ns
b	5 µm diameter % score 3	6	ultrasoft vs soft	0.00005811	****
b	5 µm diameter % score 3	6	ultrasoft vs stiff	0.0001475	***
b	5 µm diameter % score 3	6	soft vs stiff	0.262	ns
b	5 µm diameter score3/cell	6	ultrasoft vs soft	0.0036	**
b	5 µm diameter score3/cell	6	ultrasoft vs stiff	0.0023	**
b	5 µm diameter score3/cell	6	soft vs stiff	0.8587	ns
b	10 µm diameter cell number	6	ultrasoft vs soft	0.2628	ns
b	10 µm diameter cell number	6	ultrasoft vs stiff	0.0885	ns
b	10 µm diameter cell number	6	soft vs stiff	0.6827	ns
b	10 µm diameter % score3	6	ultrasoft vs soft	0.0151	*
b	10 µm diameter % score 3	6	ultrasoft vs stiff	0.00006099	****
b	10 µm diameter % score3	6	soft vs stiff	0.0007	***
b	10 µm diameter score3/cell	6	ultrasoft vs soft	0.007	**
b	10 µm diameter score3/cell	6	ultrasoft vs stiff	0.00003427	****
b	10 µm diameter score3/cell	6	soft vs stiff	0.0005	***
e	cell number	6	PDL vs PDL+laminin	0.9975	ns
e	cell number	6	PDL vs fibronectin	0.0812	ns
e	cell number	6	PDL+laminin vs fibronectin	0.0885	ns
e	% score 3	6	PDL vs PDL+laminin	0.0111	*
e	% score 3	6	PDL vs fibronectin	0.3797	ns
e	% score 3	6	PDL+laminin vs fibronectin	0.0591	ns
e	score3/cell	6	PDL vs PDL+laminin	0.1523	ns
e	score3/cell	6	PDL vs fibronectin	0.0137	*
e	score3/cell	6	PDL+laminin vs fibronectin	0.1939	ns
h	cell number	8	ultrasoft-PDL vs ultrasoft-PDL+laminin	0.9013	ns
h	cell number	8	ultrasoft-PDL vs stiff-PDL	0.8393	ns
h	cell number	8	ultrasoft-PDL+laminin vs stiff-PDL+laminin	0.9953	ns
h	cell number	8	stiff-PDL vs stiff-PDL+laminin	0.599	ns
h	% score 3	8	ultrasoft-PDL vs ultrasoft-PDL+laminin	0.0284	*
h	% score 3	8	ultrasoft-PDL vs stiff-PDL	0.0029	**
h	% score 3	8	ultrasoft-PDL+laminin vs stiff-PDL+laminin	0.003	**
h	% score 3	8	stiff-PDL vs stiff-PDL+laminin	0.0295	*
h	score3/cell	8	ultrasoft-PDL vs ultrasoft-PDL+laminin	0.9988	ns
h	score3/cell	8	ultrasoft-PDL vs stiff-PDL	0.0071	**
h	score3/cell	8	ultrasoft-PDL+laminin vs stiff-PDL+laminin	0.0074	**
h	score3/cell	8	stiff-PDL vs stiff-PDL+laminin	0.9979	ns

Supplementary Table 2: Statistics derived from Fig. 4. One-way ANOVA with Tukey's tests were employed, *p < 0.05, **p < 0.01, and ***p < 0.001, ****p<0.0001.

Panel	Plot	Df	Comparison	Exact P-value	Summary
b	cell number	12	DMSO-Soft vs. DMSO-Intermediate	0.8893	ns
b	cell number	12	DMSO-Soft vs. Benz-Soft	0.1603	ns
b	cell number	12	DMSO-Soft vs. Clem-Soft	0.7694	ns
b	cell number	12	DMSO-Intermediate vs. Benz-Intermediate	0.3835	ns
b	cell number	12	DMSO-Intermediate vs. Clem-Intermediate	0.7413	ns
b	cell number	12	Benz-Soft vs. Benz-Intermediate	0.9974	ns
b	cell number	12	Benz-Soft vs. Clem-Soft	0.7694	ns
b	cell number	12	Benz-Intermediate vs. Clem-Intermediate	0.9842	ns
b	cell number	12	Clem-Soft vs. Clem-Intermediate	0.8686	ns
b	% score 3	12	DMSO-Soft vs. DMSO-Intermediate	0.0002	***
b	% score 3	12	DMSO-Soft vs. Benztropine-Soft	0.0039	**
b	% score 3	12	DMSO-Soft vs. Clemastine-Soft	0.0006	***
b	% score 3	12	DMSO-Intermediate vs. Benztropine-Intermediate	6.52E-07	****
b	% score 3	12	DMSO-Intermediate vs. Clemastine-Intermediate	0.00001173	****
b	% score 3	12	Benztropine-Soft vs. Benztropine-Intermediate	1.11E-07	****
b	% score 3	12	Benztropine-Soft vs. Clemastine-Soft	0.814	ns
b	% score 3	12	Benztropine-Intermediate vs. Clemastine-Intermediate	0.1275	ns
b	% score 3	12	Clemastine-Soft vs. Clemastine-Intermediate	0.000004869	****
b	score3/cell	12	DMSO-Soft vs. DMSO-Intermediate	0.9991	ns
b	score3/cell	12	DMSO-Soft vs. Benztropine-Soft	0.9999	ns
b	score3/cell	12	DMSO-Soft vs. Clemastine-Soft	0.009509	**
b	score3/cell	12	DMSO-Intermediate vs. Benztropine-Intermediate	0.000003325	****
b	score3/cell	12	DMSO-Intermediate vs. Clemastine-Intermediate	0.000007335	****
b	score3/cell	12	Benztropine-Soft vs. Benztropine-Intermediate	0.00000231	****
b	score3/cell	12	Benztropine-Soft vs. Clemastine-Soft	0.005319	**
b	score3/cell	12	Benztropine-Intermediate vs. Clemastine-Intermediate	0.9748	ns
b	score3/cell	12	Clemastine-Soft vs. Clemastine-Intermediate	0.002547	**
e	cell number	6	DMSO vs. GSK	0.1654	ns
e	cell number	6	DMSO vs. Simvastatin	0.5138	ns
e	cell number	6	GSK vs. Simvastatin	0.6277	ns
e	% score 3	6	DMSO vs. GSK	0.0002	***
e	% score 3	6	DMSO vs. Simvastatin	0.0001	***
e	% score 3	6	GSK vs. Simvastatin	0.4915	ns
e	score3/cell	6	DMSO vs. GSK	0.0216	*
e	score3/cell	6	DMSO vs. Simvastatin	0.0001	***
e	score3/cell	6	GSK vs. Simvastatin	0.0017	**
h	cell number	6	DMSO vs. Wiskostatin-10 µM	0.9979	ns
h	cell number	6	DMSO vs. Wiskostatin-50 µM	0.0741	ns
h	cell number	6	Wiskostatin-10 µM vs. Wiskostatin-50 µM	0.0801	ns
h	% score 3	6	DMSO vs. Wiskostatin-10 µM	0.00004825	****
h	% score 3	6	DMSO vs. Wiskostatin-50 µM	0.000004692	****
h	% score 3	6	Wiskostatin-10 µM vs. Wiskostatin-50 µM	0.0032	**
h	score3/cell	6	DMSO vs. Wiskostatin-10 µM	0.0117	*
h	score3/cell	6	DMSO vs. Wiskostatin-50 µM	0.0015	**
h	score3/cell	6	Wiskostatin-10 µM vs. Wiskostatin-50 µM	0.146	ns

Supplementary Table 3: Statistics derived from Fig. 5. One-way ANOVA with Tukey's tests were employed, $*p < 0.05$, $**p < 0.01$, and $***p < 0.001$, $****p<0.0001$.

Panel	Plot	Df	Comparison	Exact P-value	Summary
a	Score 0, 3 µm diameter	21	5 µm vs 10 µm	0.007	**
a	Score 0, 3 µm diameter	21	5 µm vs 15 µm	0.0004	***
a	Score 0, 3 µm diameter	21	10 µm vs 15 µm	0.4719	ns
a	Score 0, 5 µm diameter	21	5 µm vs 10 µm	0.0675	ns
a	Score 0, 5 µm diameter	21	5 µm vs 15 µm	0.0854	ns
a	Score 0, 5 µm diameter	21	10 µm vs 15 µm	0.9921	ns
a	Score 0, 10 µm diameter	21	5 µm vs 10 µm	0.5414	ns
a	Score 0, 10 µm diameter	21	5 µm vs 15 µm	0.1277	ns
a	Score 0, 10 µm diameter	21	10 µm vs 15 µm	0.6055	ns
a	Score 1, 3 µm diameter	21	5 µm vs 10 µm	0.941	ns
a	Score 1, 3 µm diameter	21	5 µm vs 15 µm	0.4969	ns
a	Score 1, 3 µm diameter	21	10 µm vs 15 µm	0.6984	ns
a	Score 1, 5 µm diameter	21	5 µm vs 10 µm	0.0473	*
a	Score 1, 5 µm diameter	21	5 µm vs 15 µm	0.1553	ns
a	Score 1, 5 µm diameter	21	10 µm vs 15 µm	0.8117	ns
a	Score 1, 10 µm diameter	21	5 µm vs 10 µm	0.4645	ns
a	Score 1, 10 µm diameter	21	5 µm vs 15 µm	0.0936	ns
a	Score 1, 10 µm diameter	21	10 µm vs 15 µm	0.584	ns
a	Score 2, 3 µm diameter	21	5 µm vs 10 µm	0.8905	ns
a	Score 2, 3 µm diameter	21	5 µm vs 15 µm	0.9908	ns
a	Score 2, 3 µm diameter	21	10 µm vs 15 µm	0.9417	ns
a	Score 2, 5 µm diameter	21	5 µm vs 10 µm	0.4017	ns
a	Score 2, 5 µm diameter	21	5 µm vs 15 µm	0.855	ns
a	Score 2, 5 µm diameter	21	10 µm vs 15 µm	0.7181	ns
a	Score 2, 10 µm diameter	21	5 µm vs 10 µm	0.9991	ns
a	Score 2, 10 µm diameter	21	5 µm vs 15 µm	0.5715	ns
a	Score 2, 10 µm diameter	21	10 µm vs 15 µm	0.5963	ns
b	Score 0, 5 µm distance	21	3 µm vs 5 µm	0.03167	*
b	Score 0, 5 µm distance	21	3 µm vs 10 µm	0.0002724	***
b	Score 0, 5 µm distance	21	5 µm vs 10 µm	0.1223	ns
b	Score 0, 10 µm distance	21	3 µm vs 5 µm	0.01192	*
b	Score 0, 10 µm distance	21	3 µm vs 10 µm	0.001912	**
b	Score 0, 10 µm distance	21	5 µm vs 10 µm	0.7123	ns
b	Score 0, 15 µm distance	21	3 µm vs 5 µm	0.3746	ns
b	Score 0, 15 µm distance	21	3 µm vs 10 µm	0.01455	*
b	Score 0, 15 µm distance	21	5 µm vs 10 µm	0.2193	ns
b	Score 1, 5 µm distance	21	3 µm vs 5 µm	0.9966	ns
b	Score 1, 5 µm distance	21	3 µm vs 10 µm	0.02248	*
b	Score 1, 5 µm distance	21	5 µm vs 10 µm	0.02669	*
b	Score 1, 10 µm distance	21	3 µm vs 5 µm	0.04087	*
b	Score 1, 10 µm distance	21	3 µm vs 10 µm	0.000188	***
b	Score 1, 10 µm distance	21	5 µm vs 10 µm	0.07185	ns
b	Score 1, 15 µm distance	21	3 µm vs 5 µm	0.9258	ns
b	Score 1, 15 µm distance	21	3 µm vs 10 µm	0.007251	**
b	Score 1, 15 µm distance	21	5 µm vs 10 µm	0.01689	*
b	Score 2, 5 µm distance	21	3 µm vs 5 µm	0.9704	ns
b	Score 2, 5 µm distance	21	3 µm vs 10 µm	0.276	ns
b	Score 2, 5 µm distance	21	5 µm vs 10 µm	0.1895	ns
b	Score 2, 10 µm distance	21	3 µm vs 5 µm	0.3535	ns
b	Score 2, 10 µm distance	21	3 µm vs 10 µm	0.05851	ns
b	Score 2, 10 µm distance	21	5 µm vs 10 µm	0.5652	ns
b	Score 2, 15 µm distance	21	3 µm vs 5 µm	0.9499	ns
b	Score 2, 15 µm distance	21	3 µm vs 10 µm	0.04234	*
b	Score 2, 15 µm distance	21	5 µm vs 10 µm	0.07857	ns

Supplementary Table 4: Statistics derived from Extended Data Fig. 4. One-way ANOVA with Tukey's tests were employed, *p < 0.05, **p < 0.01, and ***p < 0.001, ****p<0.0001.

Panel	Plot	Df	Comparison	Exact P-value	Summary
a	5 µm diameter, score 0	6	Ultrasoft vs. Soft	0.0001271	***
a	5 µm diameter, score 0	6	Ultrasoft vs. Stiff	0.000117	***
a	5 µm diameter, score 0	6	Soft vs. Stiff	0.9879	ns
a	10 µm diameter, score 0	6	Ultrasoft vs. Soft	0.0001631	***
a	10 µm diameter, score 0	6	Ultrasoft vs. Stiff	0.0000133	****
a	10 µm diameter, score 0	6	Soft vs. Stiff	0.004702	**
a	5 µm diameter, score 1	6	Ultrasoft vs. Soft	0.0002175	***
a	5 µm diameter, score 1	6	Ultrasoft vs. Stiff	0.0007104	***
a	5 µm diameter, score 1	6	Soft vs. Stiff	0.2532	ns
a	10 µm diameter, score 1	6	Ultrasoft vs. Soft	0.2028	ns
a	10 µm diameter, score 1	6	Ultrasoft vs. Stiff	0.4785	ns
a	10 µm diameter, score 1	6	Soft vs. Stiff	0.04289	*
a	5 µm diameter, score 2	6	Ultrasoft vs. Soft	0.018	*
a	5 µm diameter, score 2	6	Ultrasoft vs. Stiff	0.4089	ns
a	5 µm diameter, score 2	6	Soft vs. Stiff	0.09485	ns
a	10 µm diameter, score 2	6	Ultrasoft vs. Soft	0.003232	**
a	10 µm diameter, score 2	6	Ultrasoft vs. Stiff	0.01742	*
a	10 µm diameter, score 2	6	Soft vs. Stiff	0.0001801	***
b	% score 0	6	PDL vs. PDL-Laminin	0.0671	ns
b	% score 0	6	PDL vs. Fibronectin	0.9925	ns
b	% score 0	6	PDL-Laminin vs. Fibronectin	0.058	ns
b	% score 1	6	PDL vs. PDL-Laminin	0.5912	ns
b	% score 1	6	PDL vs. Fibronectin	0.6552	ns
b	% score 1	6	PDL-Laminin vs. Fibronectin	0.9929	ns
b	% score 2	6	PDL vs. PDL-Laminin	0.0845	ns
b	% score 2	6	PDL vs. Fibronectin	0.2396	ns
b	% score 2	6	PDL-Laminin vs. Fibronectin	0.7042	ns
c	% score 0	8	Ultrasoft-PDL vs. Ultrasoft-PDL+Laminin	0.056	ns
c	% score 0	8	Ultrasoft-PDL vs. Stiff-PDL	0.0432	*
c	% score 0	8	Ultrasoft-PDL+Laminin vs. Stiff-PDL+Laminin	0.0603	ns
c	% score 0	8	Stiff-PDL vs. Stiff-PDL+Laminin	0.0781	ns
c	% score 1	8	Ultrasoft-PDL vs. Ultrasoft-PDL+Laminin	0.6834	ns
c	% score 1	8	Ultrasoft-PDL vs. Stiff-PDL	0.9928	ns
c	% score 1	8	Ultrasoft-PDL+Laminin vs. Stiff-PDL+Laminin	0.8763	ns
c	% score 1	8	Stiff-PDL vs. Stiff-PDL+Laminin	0.9994	ns
c	% score 2	8	Ultrasoft-PDL vs. Ultrasoft-PDL+Laminin	0.8659	ns
c	% score 2	8	Ultrasoft-PDL vs. Stiff-PDL	0.6188	ns
c	% score 2	8	Ultrasoft-PDL+Laminin vs. Stiff-PDL+Laminin	0.8009	ns
c	% score 2	8	Stiff-PDL vs. Stiff-PDL+Laminin	0.6967	ns

Supplementary Table 5: Statistics derived from Extended Data Fig. 5. One-way ANOVA with Tukey's tests were employed, *p < 0.05, **p < 0.01, and ***p < 0.001, ****p<0.0001.

Panel	Plot	Df	Comparison	Exact P-value	Summary
a	% score 0	12	DMSO-Soft vs. DMSO-Intermediate	0.000001319	****
a	% score 0	12	DMSO-Soft vs. Benztropine-Soft	0.000006425	****
a	% score 0	12	DMSO-Soft vs. Clemastine-Soft	7.36E-10	****
a	% score 0	12	DMSO-Intermediate vs. Benztropine-Intermediate	0.000001379	****
a	% score 0	12	DMSO-Intermediate vs. Clemastine-Intermediate	1.49E-07	****
a	% score 0	12	Benztropine-Soft vs. Benztropine-Intermediate	3.32E-07	****
a	% score 0	12	Clemastine-Soft vs. Clemastine-Intermediate	0.0862	ns
a	% score 1	12	DMSO-Soft vs. DMSO-Intermediate	0.4443	ns
a	% score 1	12	DMSO-Soft vs. Benztropine-Soft	0.4346	ns
a	% score 1	12	DMSO-Soft vs. Clemastine-Soft	0.0063	**
a	% score 1	12	DMSO-Intermediate vs. Benztropine-Intermediate	0.958	ns
a	% score 1	12	DMSO-Intermediate vs. Clemastine-Intermediate	0.9551	ns
a	% score 1	12	Benztropine-Soft vs. Benztropine-Intermediate	0.954	ns
a	% score 1	12	Clemastine-Soft vs. Clemastine-Intermediate	0.4708	ns
a	% score 2	12	DMSO-Soft vs. DMSO-Intermediate	0.6386	ns
a	% score 2	12	DMSO-Soft vs. Benztropine-Soft	0.3216	ns
a	% score 2	12	DMSO-Soft vs. Clemastine-Soft	0.000006054	****
a	% score 2	12	DMSO-Intermediate vs. Benztropine-Intermediate	0.9991	ns
a	% score 2	12	DMSO-Intermediate vs. Clemastine-Intermediate	0.0929	ns
a	% score 2	12	Benztropine-Soft vs. Benztropine-Intermediate	0.9231	ns
a	% score 2	12	Clemastine-Soft vs. Clemastine-Intermediate	0.0026	**
b	% score 0	6	DMSO vs GSK	3.67E-07	****
b	% score 0	6	DMSO vs Simvastatin	4.12E-07	****
b	% score 1	6	DMSO vs GSK	0.0004	***
b	% score 1	6	DMSO vs Simvastatin	0.0001	***
b	% score 2	6	DMSO vs GSK	0.00007371	****
b	% score 2	6	DMSO vs Simvastatin	0.0005	***
c	% score 0	6	DMSO vs Wiskostatin-10 µM	0.1761	ns
c	% score 0	6	DMSO vs Wiskostatin-50 µM	0.001093	**
c	% score 1	6	DMSO vs Wiskostatin-10 µM	0.9114	ns
c	% score 1	6	DMSO vs Wiskostatin-50 µM	0.0134	*
c	% score 2	6	DMSO vs Wiskostatin-10 µM	0.2427	ns
c	% score 2	6	DMSO vs Wiskostatin-50 µM	0.003355	**

Supplementary Table 6: Statistics derived from Extended Data Fig. 6. One-way ANOVA with Tukey's tests were employed, $*p < 0.05$, $**p < 0.01$, and $***p < 0.001$, $****p<0.0001$.
